# Hospital Meetings, &c.

**Published:** 1898-12-03

**Authors:** 


### HOSPITAL MEETINGS, &C.

### METROPOLITAN HOSPITAL SUNDAY FUND.

On Monday, November 28th, a meeting of the council of the Metropolitan Hospital Sunday Fund was held at the Mansion House, the Bishop of Southwark in the chair. The special purpose of the meeting was to determine the report of the council and to consider what day of 1899 should be recommended for next Hospital Sunday. The report stated that the year ended October 31st, 1898, the twenty-sixth year of collecting the Fund, had resulted, under the presidency of the then Lord Mayor, Sir Horatio Davies, in a total sum of £40,397. The usual collections from congregations within the metropolitan area, this year numbering 1,784, on June 12th aggregated £36,508. Among the contributors to this amount, special mention was made of the Rev. C. J. Ridgeway, of Christ Church, Lancaster Gate, who headed the list with £1,367; Rev. Canon Fleming, St. Michael's, Chester Square, £1,268; Rev. J. Storrs, St. Peter's, Eaton Square, £681; Rev. Prebendary Eardley Wilmot, St. Jude's, South Kensington, £623; and Rev. F. E. Ridgeway, St. Peter's, Cranley Gardens, £501. Sir Savile Crossley had again contributed £500; £300 had been received from A. G. P.; £200 from Mr. George Herring; while "Delta" sent his twentieth donation of £200. The working expenses for 1898 were £1,649, being 4·019 per cent. of the gross receipts, as compared with an average expenditure of £1,639, and an average percentage for 26 years of 3·859; the amount distributed being £34,304 among 132 hospitals and 54 dispensaries.

Before the report was adopted, Sir Henry Burdett suggested that there should be added to it some expression of their regret at the loss they had sustained by the death of Sir Stuart Knill. He was sure all would agree, though on occasions they might have differed from him in opinions, that he was a fine man and a noble man—a man who promoted and who stood up for all that was best in charity. Sir Henry moved, and it was seconded by Mr. Grundy, and agreed to without dissent, that the resolution of regret and condolence already passed by the council at a previous meeting should be inserted in the report, in order that it might thus be put on record in a public manner.

On the question of the revision of the list of the council for 1899, Mr. Custance, the secretary, said he had not yet received a reply from Sir Horatio Davies as to whether he would be willing to fill the vacancy caused by the death of Lord Charles Bruce; but this doubt was set at rest by Mr. Soulsby—the Lord Mayor's private secretary—who said Sir Horatio had told him he should be very happy to serve. The present Earl of Strafford's name was substituted for that of the late Earl, and it was then mooted whether as Dr. Jenkins, representing Wesleyan interests on the council, had apparently left London—letters addressed to him having been returned—he should be replaced. Sir Henry Burdett, however, appealed to the council to let the matter stand over. Dr. Jenkins was an old man, they knew, but he felt sure that none took a greater interest in the

Fund, and he should like his name to remain on the list for another year. The suggestion to remove it accordingly dropped.

Archdeacon Sinclair then proposed that June 11th next —the second Sunday in the month and the second Sunday after Trinity—be the date for next year's Hospital Sunday. That date, which moreover happened to be St. Barnabas' Day, had the approval of the Bishop of London.

The motion was adopted, with the proviso, on the suggestion of Canon Graham, that the Catholic community would, as had been done on several previous occasions, make their collection on another day, that not being a convenient one for them.

December 12th instant was decided upon for the next annual general meeting of constituents, and the meeting separated with the customary vote of thanks to the chairman for presiding.

### ADDENBROOKE'S HOSPITAL, CAMBRIDGE.

Dr. MacAlister's Resolution.—The Result of the Poll.

The recommendations of the special committee of governors of Addenbrooke's Hospital, which a fortnight ago were the subject of a long and heated discussion, are likely after all to meet with general approval. The only recommendation, it will be remembered, that was discussed at the meeting referred to was: "That a committee of management, consisting of eighteen governors, together with a chairman, be appointed annually, whose duties should include those at present assigned to the Weekly Board, the Select Governors, and the Finance Committee." Upon a division this was negatived by a single vote, and in the circumstances Dr. MacAlister (the chairman of the special committee) demanded a poll. A great amount of interest was manifested in the contest, which was therefore carried on with much activity on both sides. The polling was fixed for Monday, November 27th. By a resolution of the Weekly Board, Dr. Waraker, Dr. Besant, Mr. Spalding, Mr. Morrel, Mr. Clark, Mr. A. W. Smith, and Mr. Peck, with Mr. Bonnett (the secretary) were deputed to conduct the ballot, and there was open voting. The result was declared at ten minutes past four as follows:—

| | |
|---|---|
| For the recommendations... ... ... ... | 145 |
| Against ... ... ... ... ... ... | 95 |
| Majority for Sir Henry Burdett's proposals | 50 |

The number of governors on the roll and therefore entitled to vote was 608, of whom no fewer than 122 are ladies. Of this number exactly 240 governors went to the poll, and of these 82 were ladies.

